# Transcutaneous auricular vagus nerve stimulation regulates gut microbiota mediated peripheral inflammation and metabolic disorders to suppress depressive-like behaviors in CUMS rats

**DOI:** 10.3389/fmicb.2025.1576686

**Published:** 2025-07-30

**Authors:** Xinjiang Zhang, Xun He, Yanan Zhao, Qi Zou, Yulong Liang, Jinling Zhang, Bowen Feng, Peijing Rong, Yu Wang

**Affiliations:** ^1^Institute of Acupuncture and Moxibustion, China Academy of Chinese Medical Sciences, Beijing, China; ^2^Institute of Basic Research in Clinical Medicine, China Academy of Chinese Medical Sciences, Beijing, China

**Keywords:** depression, gut microbiota, metabolomics, brain-gut axis, transcutaneous auricular vagus nerve stimulation

## Abstract

**Background:**

Depression is a common mental disorder, and the changes of intestinal microflora and peripheral plasma metabolites can affect the gut-brain axis through vagus nerve, leading to the occurrence, and progress of the disease. Transcutaneous auricular vagus nerve stimulation (taVNS) has been previously shown to be clinically safe and effective in treating depression. However, there is no evidence whether its antidepressant effect is related to the regulation of intestinal flora and metabolites.

**Objective:**

This study investigated the gut microbiota and plasma metabolism mechanisms of taVNS in the treatment of depression.

**Methods:**

In this study, we established a chronic unpredictable mild stress (CUMS) model in SD rats for 5 weeks. During the last 3 weeks of CUMS treatment, the rats received continuous taVNS intervention for 3 weeks. Depressive-like behavior in SD rats was evaluated through behavioral assessments. The gut microbiota and plasma were analyzed using 16S rRNA gene sequencing and liquid chromatography-mass spectrometry (LC-MS) techniques.

**Results:**

Behavioral tests showed that taVNS significantly reversed the depressive-like behavior induced by CUMS in rats. 16S rRNA sequencing results showed that taVNS could improve the intestinal flora structure of CUMS rats. Microbial community characterization index showed that taVNS could reverse the gut microbiota dysbiosis in CUMS rats. ROC analysis revealed that Lachnospiraceae_NK4A136_group. *Parabacteroides* and Corynebacterium_1 are potential biomarkers for diagnosing gut microbiota dysbiosis in CUMS rats and could also serve as potential therapeutic targets for taVNS. Plasma metabolomics results showed that the differential metabolites between the CUMS group and the control group were primarily enriched in pathways such as bile acid metabolism, arachidonic acid metabolism and ether lipid metabolism. The differential metabolites between the taVNS group and the CUMS group were primarily enriched in pathways related to vitamin digestion and absorption, glycerophospholipid metabolism and amino acid metabolism. Correlation analysis between the gut microbiota and plasma metabolites suggested that pathogenic microbial genera such as *Lachnospiraceae, Lactobacillus*, and *Tyzzerella* were positively correlated with plasma metabolites during inflammation, bile acid, and lipid metabolism dysregulation, while beneficial microbiota showed the opposite trend.

**Conclusion:**

This study demonstrated that taVNS can regulate the gut microbiota, including *Lachnospiraceae, Lactobacillus, Tyzzerella*, and *Bacteroides* genera, which mediate peripheral inflammation, bile acid, and lipid metabolism dysregulation, thereby reversing the depressive-like behavior induced by CUMS in rats and exerting an antidepressant effect.

## Introduction

Depression is a prevalent mental disorder and a leading cause of disability worldwide, affecting an estimated 300 million individuals globally, which constitutes ~5% of the global adults (World Health Organization, 2023). Although it is now recognized that the pathogenesis of depression involves genetic, neurobiological, and psychosocial-cultural factors, the underlying mechanisms, which interact through multiple pathways, have yet to be fully elucidated (Wang H. Q. et al., 2021). Chronic stress is one of the key environmental risk factors for the onset of depression. The dysregulation of inflammatory cytokine release triggered by stress leads to neuronal damage, which through its impact on neurotransmitter transmission and neuroendocrine regulation, collectively influences the pathological process of depression (Liu et al., 2017; Yang et al., 2017). Therefore, establishing animal models of depression is a critical step in studying the specific mechanisms of depression. The most widely utilized model is the CUMS animal model, which involves the repeated application of various unpredictable mild stressors over a period of several weeks (typically 2–6 weeks) to recapitulate depressive-like phenotypes analogous to those induced by chronic stress in humans (Antoniuk et al., 2019). Moreover, recent studies have shown that the gut microbiota, through the gut-brain axis, plays a role in influencing the onset and progression of depression (Rogers et al., 2016). As a crucial component of the biochemical barrier in the gut, the gut microbiota has long been a focus of widespread attention in neuroscience for its important role in maintaining gut-brain axis homeostasis. The microbiota and the brain communicate through various pathways, including the immune system, tryptophan metabolism, the vagus nerve, and the enteric nervous system, involving microbial metabolites such as short-chain fatty acids, branched-chain amino acids, and peptidoglycans (Cryan et al., 2019). Stress can significantly alter the diversity, composition, and distribution characteristics of the gut microbiota (Ge et al., 2021). Dysbiosis of the gut microbiota is associated with an increase in pathogenic microorganisms and their related metabolites, while also activating neurotoxic substances such as inflammasomes. These changes affect the central nervous system by altering neurotransmitters through the blood-brain barrier and the vagus nerve, leading to a range of emotional and cognitive behavioral disorders (Rutsch et al., 2020). Therefore, correcting gut microbiota dysbiosis and maintaining gut-brain axis homeostasis represent potential therapeutic approaches for depression. Exploring the changes in gut microbiota and their metabolites in depression, and developing corresponding microbial therapeutic strategies, is an important scientific challenge currently facing research in this field.

Vagus nerve-mediated cholinergic signals control immune function and pro-inflammatory responses through the inflammatory reflex, playing an important role in regulating metabolic homeostasis (Pavlov and Tracey, 2012). Transcutaneous auricular vagus nerve stimulation (taVNS), as a simple and non-invasive neuromodulation therapy, has gradually gained widespread recognition for its efficacy in treating depression and other neuropsychiatric disorders. However, the biological mechanisms underlying taVNS in the treatment of depression remain to be fully elucidated. Numerous clinical and basic studies have confirmed its significant antidepressant effects, and speculated that its mechanism may involve the regulation of gut microbiota, alleviation of systemic inflammation and correction of metabolic dysregulation (Wang Y. et al., 2021). However, there is no evidence whether its antidepressant effect is related to the regulation of intestinal flora and metabolites. In our previous studies, we have not only confirmed that taVNS can significantly suppress the depressive symptoms in patients with depression (Li et al., 2022; Rong et al., 2016), but also demonstrated that it can reduce the levels of inflammation in the hippocampus and hypothalamus of CUMS rats, thereby exerting an antidepressant effect (Wang J. Y. et al., 2021; Chen et al., 2023). Gastrointestinal diseases and metabolic disorders are often accompanied by depressive symptoms. In our previous studies, we conducted a series of animal experiments on taVNS regulation of the brain-gut axis. We found that both stresses combined with iodoacetamide-induced functional dyspepsia and high-fat diet-induced diabetic rat models exhibited depressive-like behavior. In ZDF rats, which exhibit both glucose-lipid metabolism dysregulation and changes in gut microbiota, taVNS was shown to improve gastrointestinal function, restore glucose-lipid metabolism balance, and inhibit central nervous system inflammation, thereby exerting antidepressant effects in metabolic disease animal models (Li et al., 2024; Han et al., 2022). However, the changes in gut microbiota and plasma metabolic levels in depression, as well as the mechanisms through which taVNS intervenes in the gut microbiota, still require further elucidation.

In this study, we observed the effects of taVNS intervention on the gut microbiota and plasma metabolism in CUMS rats. We identified that CUMS leads to gut microbiota dysbiosis and plasma metabolite disturbances, revealing the potential roles of gut microbiota and plasma metabolites in depressive-like behaviors in rats. We also aimed to elucidate the biological mechanisms by which taVNS exert its antidepressant effects through the modulation of the gut microbiota and plasma metabolic networks.

## Materials and methods

### Experimental animals and groups

All protocols were implemented in accordance with the Guidelines for the Care and Use of Laboratory Animals of the Ministry of Science and Technology of China and approved by the Ethics Committee of the Institute of Acupuncture and Moxibustion, China Academy of Chinese Medical Sciences (No. D2019-02-11-1). Specific pathogen Free (SPF) male Sprague-Dawley (SD) rats were purchased from SPF (Beijing) Biotechnology Co., LTD. [License No. SCXK (Beijing) 2016-0002]. All rats were kept at a temperature of 22 ± 2°C with 12 h light/12 h dark cycles, and food and water were provided ad libitum. After adaptation for 1 week, the rats were randomly divided into normal group (*n* = 6), CUMS group (*n* = 6), and CUMS + taVNS group (*n* = 6).

### Chronic unpredictable mild stress models

The CUMS procedure was performed for induction of depression in a rat model as previously described (Willner, 1997) with minor modifications. CUMS protocol involves a variety of mild stressors and lasted for 35 days, including swimming in cold water (4°C, 5 min), food deprivation for 24 h, water deprivation for 24 h, clip tail for 3 min, moist bedding (24 h), 45°C cage tilting (12 h), a reversed light–dark circadian cycle (12:12 h), and restraining (6 h). One of these stressors were performed every day in a random order for rats, and each stressor would not be arranged 2 days in a row to avoid a rat predicting it.

### Intervention of taVNS

**Figure 1:**
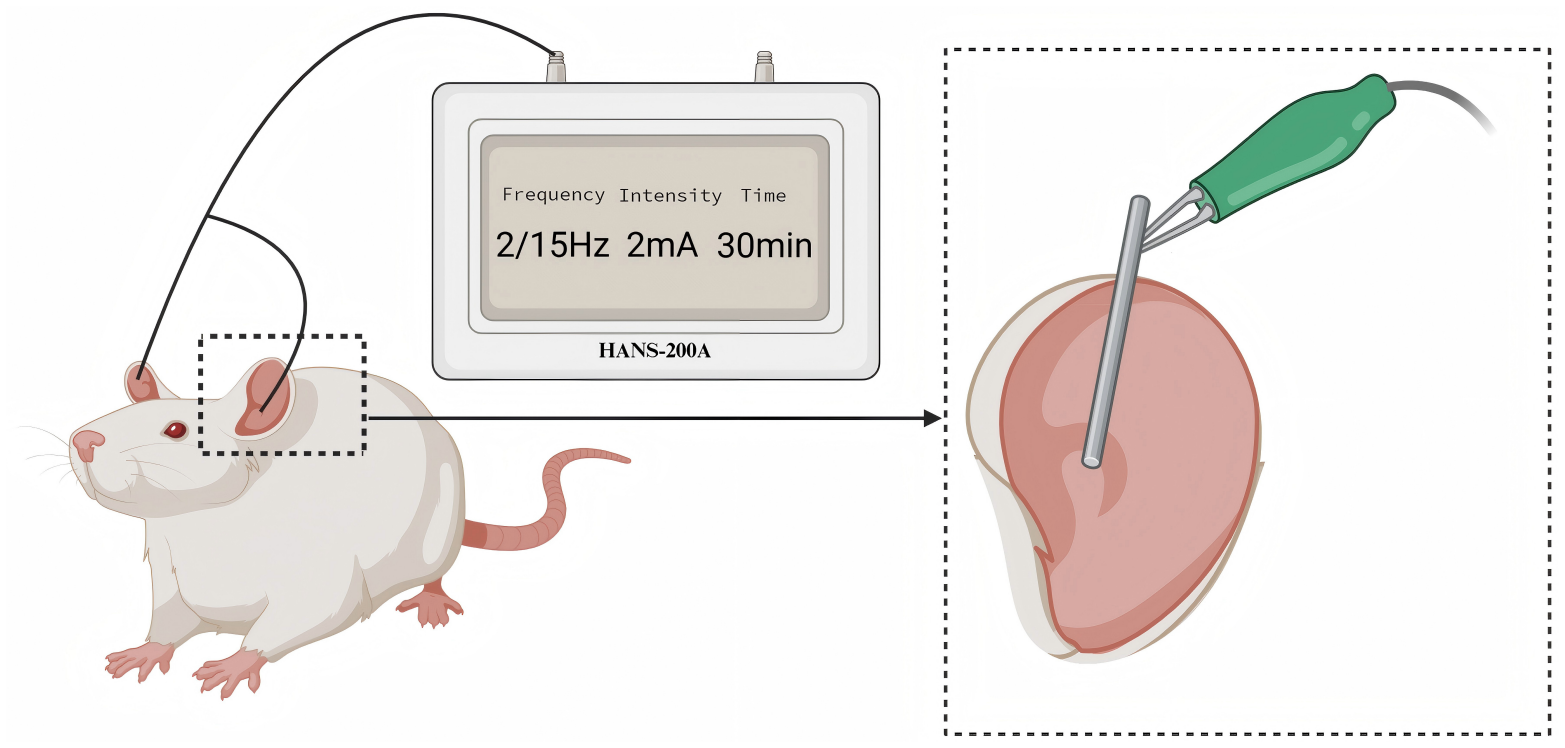
The diagram of transcutaneous auricular vagus nerve stimulation. The electrical stimulation was applied to the auricular branch of the vagus nerve distribution area, as indicated in the enlarged part of the above figure.

The protocol of taVNS was performed based on a previous study (Wang J. Y. et al., 2021). Rats were placed in a bench animal anesthesia ventilator system (VME, Matrix) and inhaled isoflurane with a 2% concentration. The positive and negative self-adsorbent conducting magnets were non-invasitively connected and fixed on both sides of the auricular nail cavities of rats and connected to an electrical stimulator (HANS-200A, Nanjing Jisheng Medical Technology Co., Ltd.). The stimulation parameters were as follows: the stimulation frequency was 2/15 Hz (2 and 15 Hz, switching once per second), the stimulation intensity was 2 mA, and the intervention lasted 30 min per day for 21 days. The effect of electrical stimulation is assessed by slight vibration of the auricle. Use a cotton swab dipped in saline to wipe the auricle to enhance the conductive effect of the electrode. The intervention time starts from 13:00–15:00 every afternoon (Figure 1).

### Body mass

In this study, the body mass of rats was weighed before modeling, after modeling, and after intervention, respectively. Body mass, an important indicator for judging whether the rat model is successful and whether the intervention is effective.

### Behavioral tests

#### Sucrose preference test

The SPT was conducted before modeling, after modeling, and after intervention, respectively. Before SPT, the rats were trained to drink 1% sucrose solution for 24 h, and then were given a bottle of 1% sucrose solution and a bottle of pure water for 24 h. After the end of the training period, the rats were deprived of water for 24 h, and the consumption of a bottle of 1% sucrose solution and a bottle of pure water within 1 h was measured for every rat. The percentage difference in sucrose preference is calculated as follows: sucrose preference ratio (%) = sucrose consumption/(sucrose consumption + pure water consumption) ×100% (Li et al., 2021).

#### Open field test

OFT of rats was carried out before modeling, after modeling, and after intervention, respectively. The rats were placed in the center of the bottom of the open field box (100 cm × 100 cm × 40 cm black chamber with 25 squares of the same size marked with white lines), and the rats were free to explore in the box for 5 min. Before the test, the inner wall and bottom of the open field box were wiped with 75% alcohol to avoid interference with the biological information left by the rats in the previous round of test (Li et al., 2020). The activity of each rat was monitored and analyzed through a video analysis system (Shanghai Jiliang Software Technology Co., Ltd.).

#### Force swimming test

FST of rats was carried out before modeling, after modeling, and after intervention, respectively. The day before the test, the rats were placed in an open cylindrical container of water with a diameter of 30 cm and a height of 46 cm for swimming acclimatization for 15 min. The test will be conducted 24 h later. The rats were forced to swim under the same conditions for 5 min, and the total immobile time during the next 3 min of testing was recorded with a camera (Porsolt et al., 1977; Wang J. Y. et al., 2021).

#### Collection of fecal and blood samples

After successful modeling and intervention, rats were anesthetized by intraperitoneal injection of 3% pentobarbital sodium (0.15 ml/100 g), and blood was collected from the abdominal aorta. Blood samples and fecal pellets from the rectum were collected immediately and frozen at −80°C for subsequent gut microbiome sequencing and plasma metabolomics.

#### 16S rRNA analysis of fecal samples

Microbial DNA was extracted from fecal samples using the E.Z.N.A.^®^ soil DNA Kit (Omega Bio-tek, Norcross, GA, U.S.) according to manufacturer's protocols. The final DNA concentration and purification were determined by NanoDrop 2000 UV-vis spectrophotometer (Thermo Scientific, Wilmington, USA). The V3-V4 hypervariable regions of the bacteria 16S rRNA gene were amplified with primers 338F (5′- ACTCCTACGGGAGGCAGCAG-3′) and 806R (5′-GGACTACHVGGGTWTCTAAT-3′) by thermocycler PCR system (GeneAmp 9700, ABI, USA). Purified amplicons were pooled in equimolar and paired-end sequenced (2 × 300) on an Illumina MiSeq platform (Illumina, San Diego, USA) according to the standard protocols by Majorbio Bio-Pharm Technology Co. Ltd. (Shanghai, China). Raw fastq files were demultiplexed, quality-filtered by Trimmomatic and merged by FLASH. Operational taxonomic units (OTUs) were clustered with 97% similarity cutoff using UPARSE(version 7.1 http://drive5.com/uparse/) and chimeric sequences were identified and removed using UCHIME. The taxonomy of each 16S rRNA gene sequence was analyzed by RDP Classifier algorithm (http://rdp.cme.msu.edu/) against the Silva (SSU123) 16S rRNA database using confidence threshold of 70%. Based on OTU and its abundance results, the alpha-diversity index was calculated and plotted using QIIME software and R (v3.3.1) software, respectively. For beta diversity, weighted UniFrac distances were calculated and principal coordinate analysis (PCoA) plots were generated, and PLS-DA plots were drawn using R (v3.3.1) software. Circos-0.67-7 software was used to show the distribution proportion of each dominant species in different groups. Multilevel species difference discriminant analysis of Lefse was used to find microbial groups of different levels (α < 0.05 and LDA >3.0) that had significant influence on the differences between groups.

#### Metabolomics analysis of plasma

100 μL sample was added to a 1.5 mL centrifuge tube with 400 μL solution [acetonitrile: methanol = 1:1(v:v)] containing 0.02 mg/mL internal standard (L-2-chlorophenylalanine) to extract metabolites. The samples were mixed by vortex for 30 s and low-temperature sonicated for 30 min (5°C, 40 KHz). The samples were placed at −20°C for 30 min to precipitate the proteins. Then the samples were centrifuged for 15 min (4°C, 13,000 g). The supernatant was removed and blown dry under nitrogen. The sample was then re-solubilized with 100 μL solution (acetonitrile: water = 1:1) and extracted by low-temperature ultrasonication for 5 min (5°C, 40 KHz), followed by centrifugation at 13,000 g and 4°C for 10 min. The supernatant was transferred to sample vials for LC-MS/MS analysis. Metabolites of all samples of equal volume were mixed to prepare Quality control samples (QC). The LC-MS/MS analysis of sample was conducted on a Thermo UHPLC-Q Exactive HF-X system equipped with an ACQUITY HSS T3 column (100 mm × 2.1 mm i.d., 1.8 μm; Waters, USA) at Majorbio Bio-Pharm Technology Co. Ltd. (Shanghai, China). The mass spectrometric data were collected using a Thermo UHPLC-Q Exactive HF-X Mass Spectrometer equipped with an electrospray ionization (ESI) source operating in positive mode and negative mode.

The UHPLC-MS raw data were converted into the common format by Progenesis QI software (Waters, Milford, USA) through baseline filtering, peak identification, peak integral, retention time correction, and peak alignment. At the same time, the metabolites were identified by searching database, and the main databases were the HMDB (http://www.hmdb.ca/), Metlin (https://metlin.scripps.edu/) and the self-compiled Majorbio Database (MJDB) of Majorbio Biotechnology Co., Ltd. (Shanghai, China). The data matrix obtained by searching database was uploaded to the Majorbio cloud platform (https://cloud.majorbio.com) for data analysis. Then the R package “ropls” (Version 1.6.2) was used to perform principal component analysis (PCA) and orthogonal least partial squares discriminant analysis (OPLS-DA), and 7-cycle interactive validation evaluating the stability of the model. The metabolites with VIP >1, *p* < 0.05 were determined as significantly different metabolites based on the variable importance in the projection (VIP) obtained by the OPLS-DA model and the *p-value* generated by student's *t*-test. Differential metabolites among two groups were mapped into their biochemical pathways through metabolic enrichment and pathway analysis based on KEGG database (http://www.genome.jp/kegg/).

### Statistical analysis

Quantitative data were expressed as mean ± standard deviation. Statistical analysis and data visualization were performed using GraphPad Prism 8.0 and R (v3.3.1). Two-factor analysis of variance was used for body weight and behavioral data. During the analysis of gut microbiota and plasma metabolomics, two-tailed *T*-test (unpaired) was used to compare the difference between the two groups for normally distributed data, and Wilcoxon rank sum tests were used if the variables were inconsistent with the normal distribution. Kruskal-Wallis rank sum test was used to compare the differences among groups, and Spearman's rank correlation coefficient was used for correlation analysis. *P* < 0.05 was considered statistically significant.

## Results

### Effects of taVNS on body weight and depressive-like behaviors in CUMS rats

**Figure 2:**
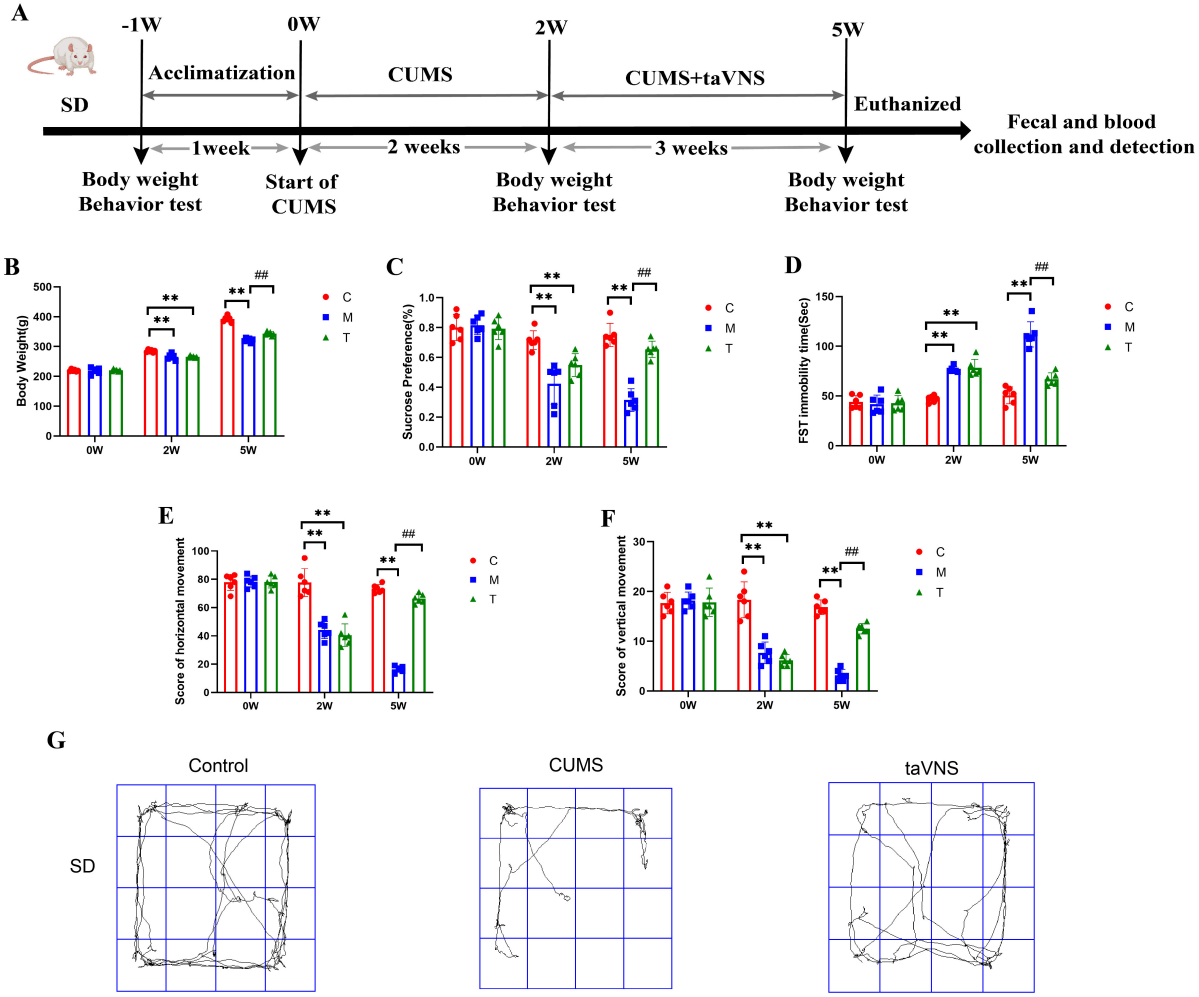
Effects of taVNS on body weight and depressive-like behaviors in CUMS rats (*n* = 6). **(A)** Schematic diagram of CUMS model establishment and schematic diagram of taVNS intervention. **(B)** Comparison of body weight at different time points. **(C)** Sucrose preference rate at different time points. **(D)** Comparison of immobility time of forced swimming at different time points. **(E)** Comparison of horizontal motion scores at different time points. **(F)** Comparison of vertical motion scores at different time points. **(G)** Representative activity traces of subjects in different groups for SD rats in the open field test. Significant group effects were observed for body weight (*F* = 92.6), sucrose preference test (*F* = 38.35), forced swimming test (*F* = 63.97), horizontal movement distances (*F* = 126.4), and vertical movement distances (*F* = 67.62). Compared with control group, **p < 0.01; Compared with CUMS group, ^*##*^*p* < 0.01. C, Control group; M, Model group (CUMS group); T, Intervention group (taVNS group).

To investigate the effects of taVNS on CUMS rats, depressive-like behavior was assessed by weight measurement, sucrose preference test, open field test, and forced swimming test (Figure 2). taVNS significantly improved body mass and suppressed depressive-like behavior in rats.

### taVNS regulates the alpha diversity and beta diversity of intestinal microorganisms in CUMS rats

**Figure 3:**
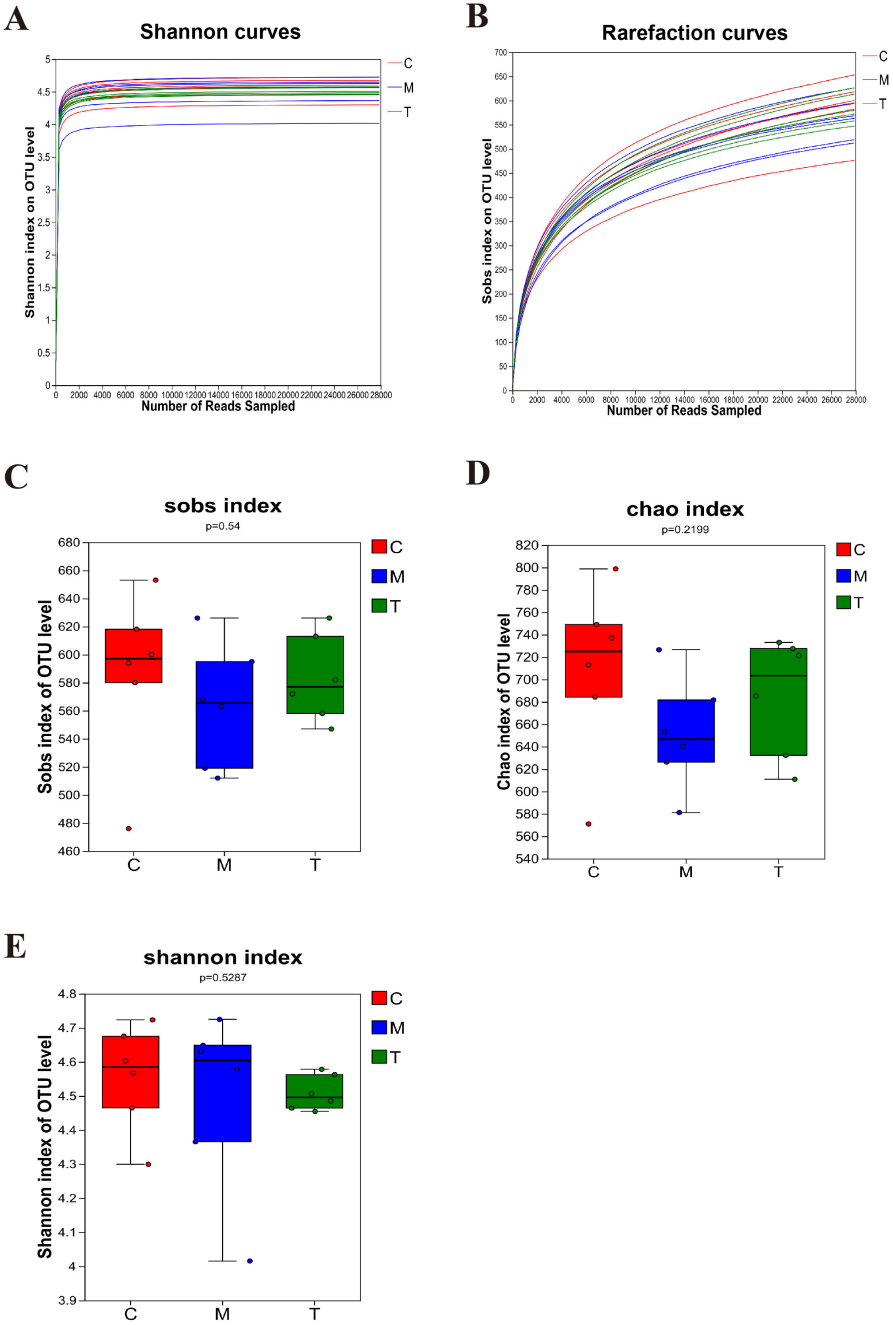
Alpha diversity of intestinal microbial samples of rats in each group (*n* = 6). **(A, B)** OTU sparse Shannon index curve and dilution curve of samples. **(C)** Sobs index. **(D)** Chao index. **(E)** Shannon index. C, Control group; M, Model group (CUMS group); T, Intervention group (taVNS group).

**Figure 4:**
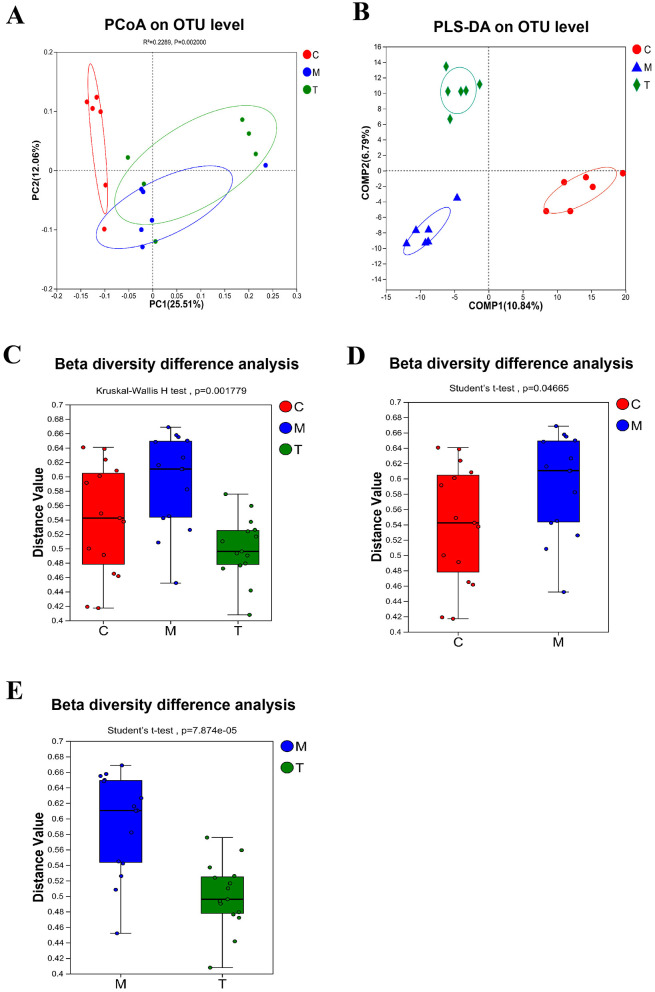
Beta diversity of intestinal microbial samples of rats in each group (*n* = 6). **(A)** PCoA analysis. **(B)** PLS-DA analysis. **(C–E)** Beta diversity difference analysis between groups. C, Control group; M, Model group (CUMS group); T, Intervention group (taVNS group).

DNA sequences of 18 fecal samples were analyzed, and dilutive curves based on sobs index (Figure 3A) and Shannone curve based on Shannon index (Figure 3B) were obtained. The diversity sparse curves of Sobs and Shannon tended to be flat, indicating that the amount of sequencing data was reasonable, the sequencing depth and sample size were sufficient, and could indirectly reflect the species abundance. Alpha diversity analysis showed that intestinal flora diversity in CUMS group decreased and increased after taVNS intervention. However, there was no significant difference between the groups (Figures 3C–E). PCoA and PLS-DA showed that samples in each group had a good degree of aggregation, with differences among groups (Figures 4A, B). Statistical analysis showed that beta diversity in CUMS group was significantly higher than that in normal control group, and significantly decreased after taVNS intervention (Figures 4C–E). The results showed that there were significant differences in community structure among the three groups, and the bacterial community structure changed significantly after taVNS intervention.

### taVNS regulates the composition of intestinal flora in CUMS rats

**Figure 5:**
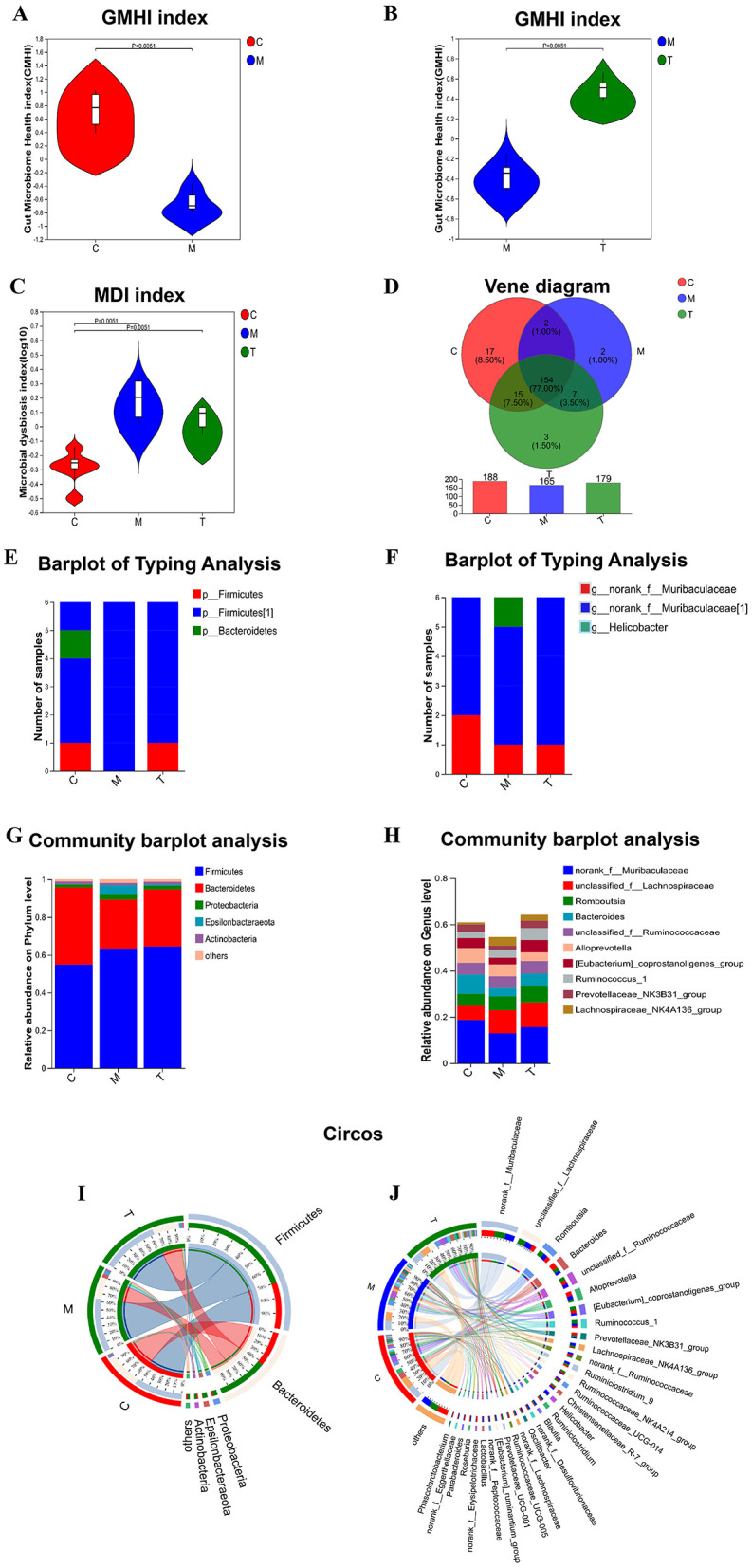
Analysis of microbial community characterization index and composition ratio (*n* = 6). **(A–C)** analysis of GMHI and MDI index; **(D)** analysis of flora in each group by Venn diagram; **(E, F)** analysis of flora typing in each group by phylum and genus level; **(G, H)** analysis of colony composition in each group by Bar diagram. **(I, J)** Circos samples and species relationship analysis of each group at phylum and genus level. C, Control group; M, Model group (CUMS group); T, Intervention group (taVNS group).

The microbial community characterization index analysis showed that the gut microbiome health index of rats in the model group was significantly lower than that in the control group (GMHIs-mean = −0.63, *P* = 0.0051), and the microbial dysbiosis index was significantly higher than that in the control group (MDI-mean=0.19, *P* = 0.0051). The disturbance of flora was significantly reversed after taVNS intervention (Figures 5A–C). We used the Venn diagram to show the difference in the number of microbiotas identified at the genus level (Figure 5D). At the phylum level, Firmicutes and Bacteroidetes are the dominant bacteria groups in the control group, while Firmicutes are the dominant bacteria groups in CUMS group and taVNS group. At the genus level, norank_f_Muribaculaceae was the dominant bacterial group in both the control group and the taVNS group, while *Helicobacter* was the dominant bacterial group in CUMS group (Figures 5E, F).

To determine the change characteristics of specific bacterial groups after taVNS intervention, we analyzed the community composition of all groups at the phylum and genus level, and selected the top 10 bacterial groups with the highest abundance to show in the form of bar chart (Figures 5G, H). Firmicutes and Bacteroidetes occupy a higher proportion in the three groups at the phylum level. Firmicutes dominate in the CUMS group and taVNS group, while Bacteroidetes dominate in the control group. Epsilonbacteraeota and Proteobacteria were dominant in the model group, and their abundance decreased after the intervention of taVNS. At the phylum and genus levels, we demonstrated the distribution ratio of the flora among the groups (Figures 5I, J).

**Figure 6:**
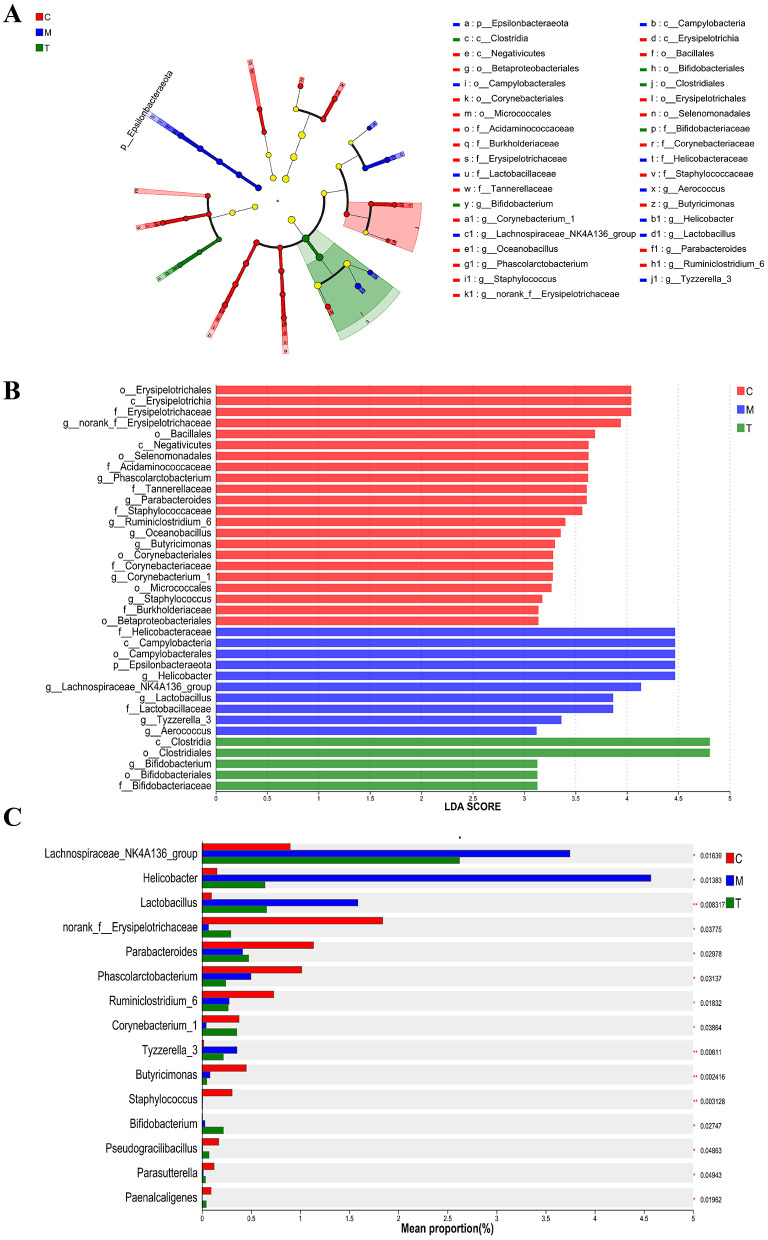
Multilevel discriminant analysis of Lefse species difference and inter-group difference test for each group (*n* = 6). **(A, B)** Branch and bar graphs generated by LEfSe analysis showed different classification levels among each group (LDA > 3.0). **(C)** Kruskal-Wallis rank sum test for the differential flora between groups. The Y-axis represents the species name, the X-axis represents the average relative abundance in different groups of species, and the far right is the *P-value*. C, Control group; M, Model group (CUMS group); T, Intervention group (taVNS group).

To identify different species of different groups between groups, Lefse multilevel discriminant analysis of species difference was used to test the difference of species at multiple levels between groups. At the generic level, norank_f_Erysipelotrichaceae, Phascolarctobacterium, *Parabacteroides*, Ruminiclostridium_6, Oceanobacillus, Butyricimonas, Corynebacterium_1, and Staphylococcus are the genera with high abundance in the normal group. *Helicobacter*, Lachnospiraceae_NK4A136_group, *Lactobacillus*, Tyzzerella_3, and Aerococcus are the genera with high abundance in CUMS group. Bifidobacterium is the most abundant genus in taVNS group (Figures 6A, B). We further screened the top 15 bacteria groups with significant differences in average abundance at genus level, among which Lachnospiraceae_NK4A136_group, *Helicobacter, Lactobacillus*, and Tyzzerella_3 were higher in CUMS group than in normal group. The abundance decreased after taVNS intervention. norank_f__Erysipelotrichaceae, *Parabacteroides*, and Corynebacterium_1 decreased in CUMS group, but increased after taVNS intervention (Figure 6C).

### Diagnosis of intestinal microbiome and ROC analysis of intervention targets in CUMS rats

**Figure 7:**
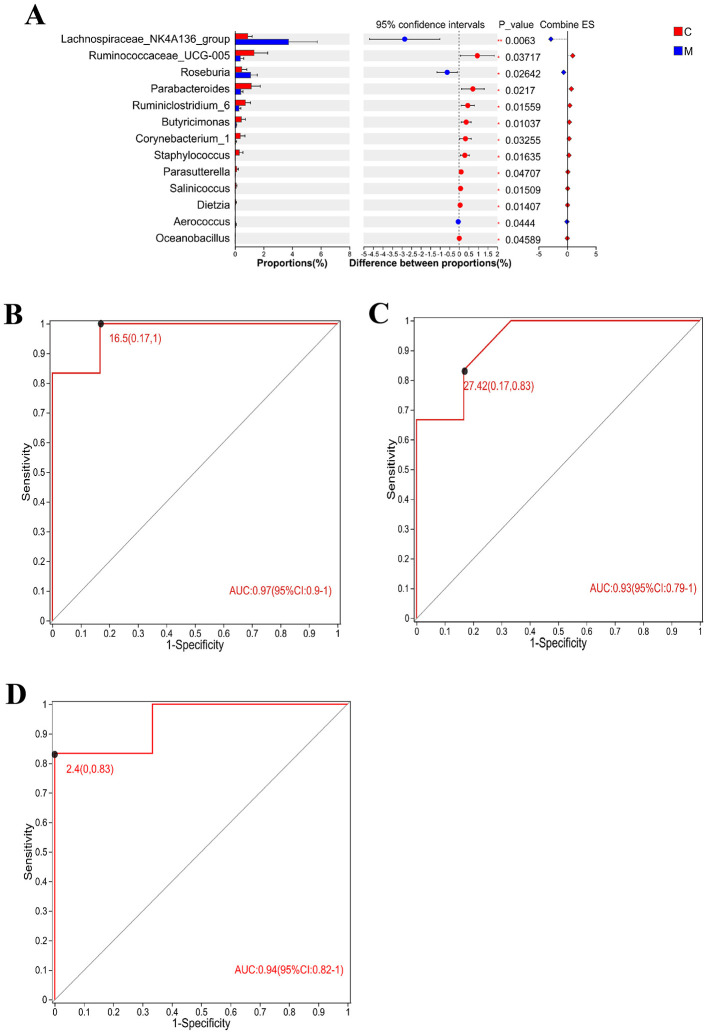
Differential flora test and ROC analysis (*n* = 6). **(A)** The *T*-test of the differential flora between the CUMS group and the blank control group formed a bar chart, in which the Y-axis represented the species name, the X-axis represented the average relative abundance of species in different groups, and the far right was the *P-value*. **(B–D)** Area under ROC curve of Lachnospiraceae_NK4A136_group, *Parabacteroides*, and Corynebacterium_1. X-axis was 1-Specificity, coordinate axis was 1–0; the Y-axis is Sensitivity and the coordinate axis is 0–1; the point marked on the curve is the optimal critical value. The AUC indicated in the figure is the area under the corresponding curve. C, blank control group; M, model group (CUMS group).

In order to identify specific microbial markers of CUMS, intestinal flora with significant differences between the control group and the model group and ranking the top 15 in average abundance (Figure 7A) were selected. In combination with the three groups difference test, Lachnospiraceae_NK4A136_group, *Parabacteroides*, and Corynebacterium_1 had significant changes in the intestinal tract of CUMS rats and could respond to taVNS intervention. ROC analysis was performed for the above three bacterial populations and ROC curves were drawn. The AUC values of Lachnospiraceae_NK4A136_group, *Parabacteroides*, and Corynebacterium_1 are 0.97 (95%CI: 0.9–1), 0.93 (95%CI: 0.79–1), and 0.94 (95%CI: 0.82–1), respectively (Figures 7B–D). Therefore, Lachnospiraceae_NK4A136_group, *Parabacteroides*, and Corynebacterium_1 are potential biomarkers of CUMS model and therapeutic targets of taVNS.

### taVNS regulates plasma metabolism in CUMS rats

**Figure 8:**
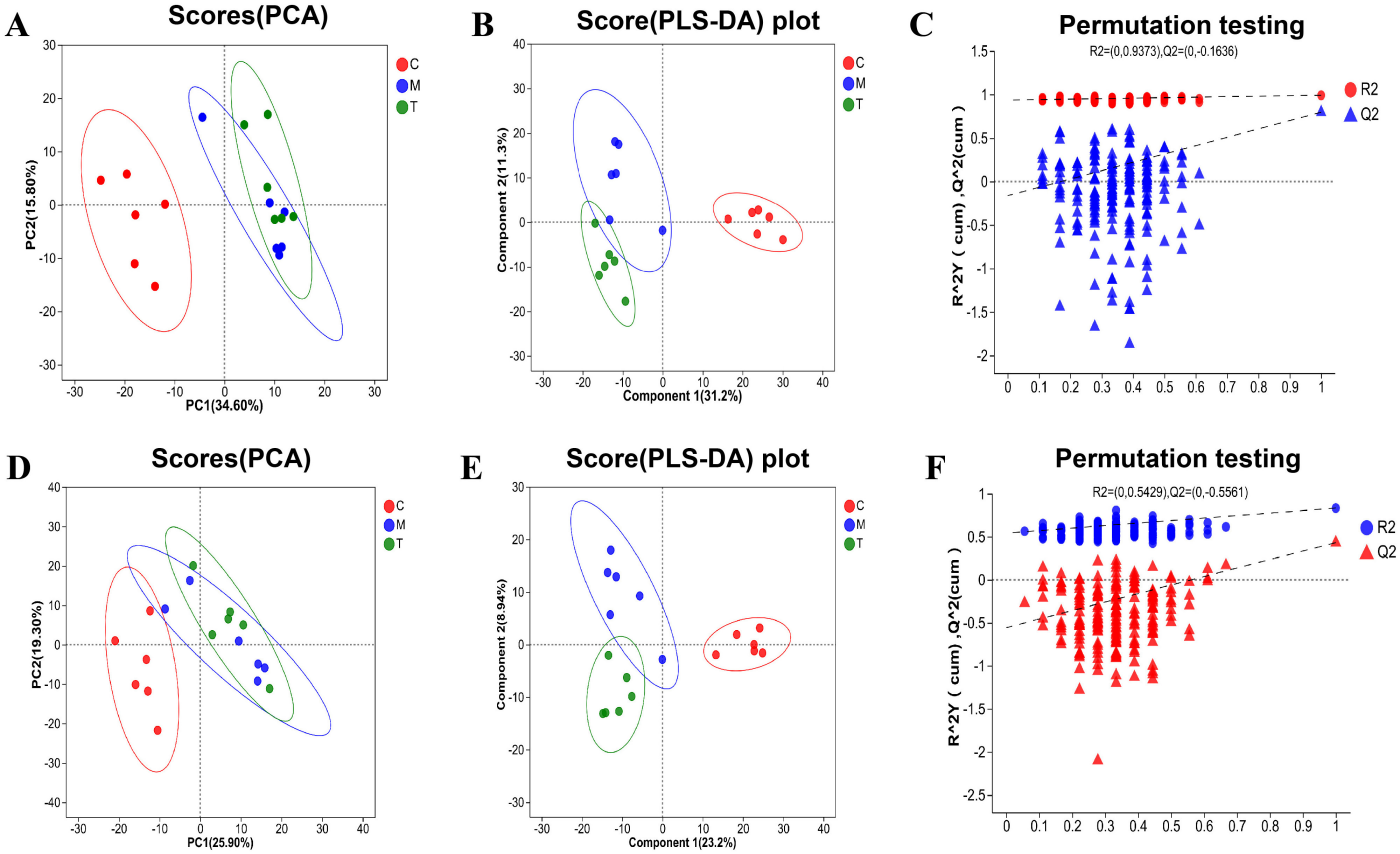
PCA and PLS-DA analysis of plasma metabolic samples from rats in each group (*n* = 6). **(A–C)** PCA and PLS-DA analysis plots in the negative ion mode. **(D–F)** PCA and PLS-DA analysis plots in the positive ion mode. C, Blank control group; M, Model group (CUMS group); T, Intervention group (taVNS group).

PCA and PLS-DA analyses demonstrated good cohesion within the sample groups. PCA analysis, as an unsupervised method, can reflect the largest sources of variation in the data, while PLS-DA, as a supervised method, is capable of capturing subtle differences, amplifying inter-group variation signals, and suppressing irrelevant noise. The group separation displayed in the PLS-DA plot shows superior clarity compared to the PCA plot (Figures 8A, B, D, E). The regression lines of R2 and Q2 showed an upward trend in the anionic and cationic modes, and the goodness-fit and predictive values of the anionic mode (R2X = 0.617, R2Y = 0.991, Q2 = 0.797) and the cationic mode (R2X = 0.321, R2Y = 0.833, Q2 = 0.433) showed that PLS-DA model has good fitting ability and effective prediction ability (Figures 8C, F).

**Figure 9:**
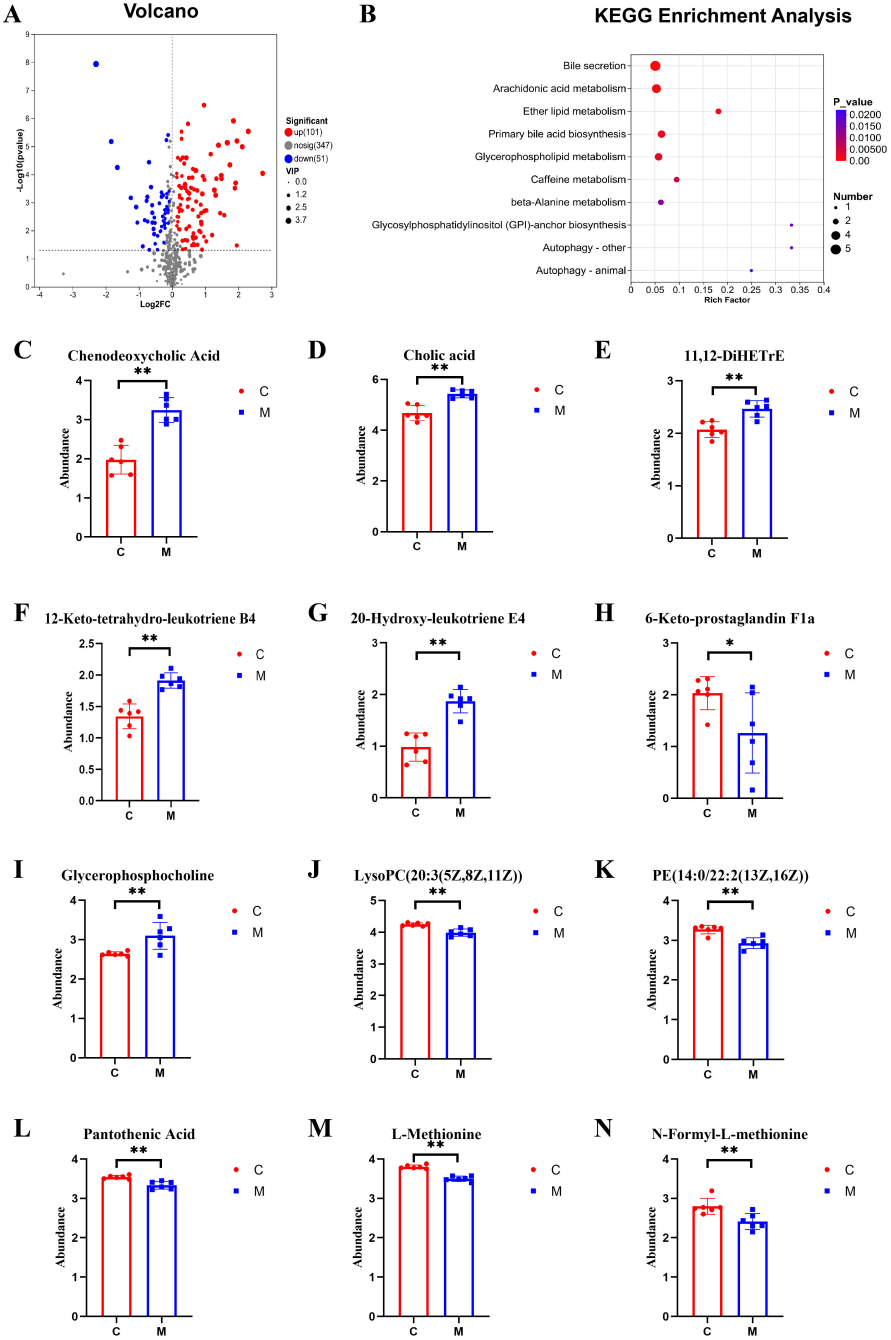
Differential metabolites between groups and KEGG pathway enrichment analysis (*n* = 6). **(A)** Volcano plot of differential metabolites between the control group and the CUMS group. **(B)** KEGG pathway enrichment bubble plot of differential metabolites between the control group and the CUMS group. **(C–N)** Bar plots of *t*-test results for differential metabolites between the control group and the CUMS group (*n* = 6). Compared with the control group, *p < 0.05, **p < 0.01. C, Control group; M, Model group (CUMS group).

**Figure 10:**
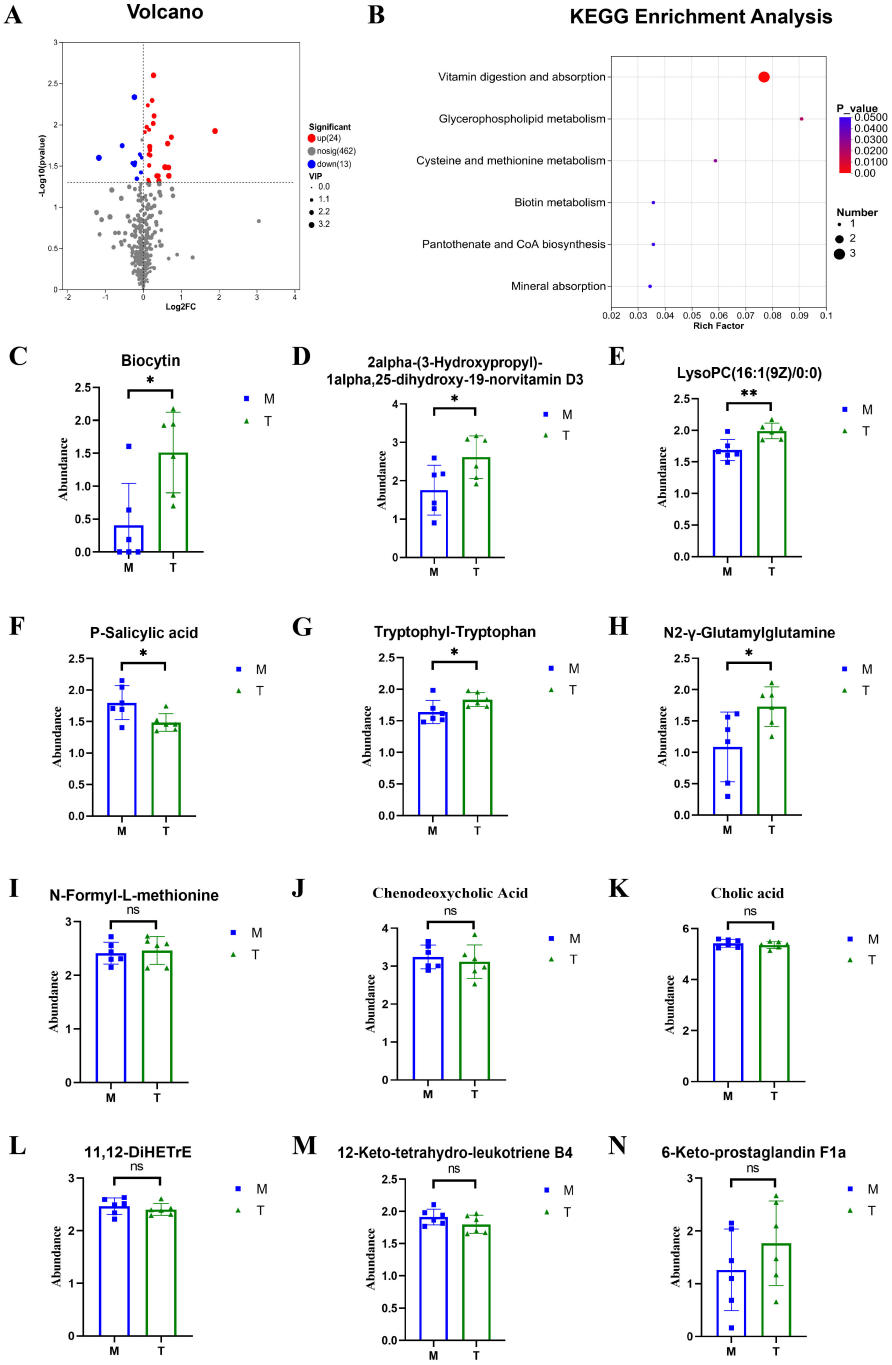
Differential metabolites between groups and KEGG pathway enrichment analysis (*n* = 6). **(A)** Volcano plot of differential metabolites between the CUMS group and the taVNS group. **(B)** KEGG pathway enrichment bubble plot of differential metabolites between the CUMS group and the taVNS group. **(C–N)** Bar plots of *t*-test results for differential metabolites between the control group and the CUMS group (*n* = 6). Compared with the CUMS group, *p < 0.05, **p < 0.01. ns, non-significant; M, Model group (CUMS group); T, Intervention group (taVNS group).

The significance of differential metabolites was analyzed by *P-value* < 0.05, VIP > 1, FC > 1 and *T*-test, and the volcano map was drawn (Figures 9A, 10A). Compared with the control group, 101 metabolites were significantly up-regulated and 51 metabolites were significantly down-regulated in CUMS group. Compared with the model group, 24 metabolites were significantly up-regulated and 13 metabolites were significantly down-regulated in taVNS group. The difference metabolites in CUMS group were significantly changed compared with the control group, and some of the difference metabolites were changed after taVNS intervention. To further analyze the plasma metabolic characteristics of CUMS rats and the effects of taVNS intervention, we conducted KEGG pathway annotation and enrichment analysis. The pathway enrichment results and the corresponding upregulated and downregulated metabolites are shown in (Figures 9B–N, 10B–N). The top five pathways with significant enrichment of differential metabolites between the control group and CUMS group were primarily involved in bile secretion, arachidonic acid metabolism, ether lipid metabolism, primary bile acid biosynthesis, and glycerophospholipid metabolism. The top five pathways with significant enrichment of differential metabolites between the CUSM group and taVNS group were primarily involved in vitamin digestion and absorption, glycerophospholipid metabolism, cysteine and methionine metabolism, pantothenate and CoA biosynthesis, and biotin metabolism.

We performed a *t*-test on the metabolites enriched in the KEGG pathways. The results showed that, compared to the control group, bile acids and arachidonic acid-related metabolites such as chenodeoxycholic acid, cholic acid, 11,12-DiHETrE, 12-keto-tetrahydro-leukotriene B4, and 20-hydroxy-leukotriene E4 were significantly upregulated in the CUMS group, while 6-Keto-prostaglandin F1a was significantly downregulated. In the lipid metabolism pathway, glycerophosphocholine was significantly upregulated in the CUMS group, while LysoPC [20:3(5Z,8Z,11Z)] and PE [14:0/22:2(13Z,16Z)] were significantly downregulated. Metabolites related to amino acid biosynthesis, such as pantothenic acid, L-Methionine, and N-Formyl-L-methionine were significantly downregulated in the CUMS group. After taVNS intervention, several metabolites showed significant changes. For instance, biologically active substances related to vitamin digestion and absorption, such as biocytin and 2alpha-(3-Hydroxypropyl)-1alpha, 25-dihydroxy-19-norvitamin D3 were significantly upregulated. In the lipid metabolism pathway, LysoPC (16:1(9Z)/0:0) was significantly upregulated. P-Salicylic acid related to the amino acid metabolism was significantly downregulated. Metabolites related to amino acid biosynthesis, such as tryptophanyl-tryptophan and N2-γ-glutamine, were significantly upregulated, while N-Formyl-L-methionine was upregulated but showed no statistical difference. Metabolites related to bile acids and arachidonic acid, such as chenodeoxycholic acid, cholic acid, 11,12-DiHETrE, and 12-keto-tetrahydro-leukotriene B4, showed a downward trend, and 6-Keto-prostaglandin F1a showed an upward trend.

### Correlation analysis between gut microbiota and plasma metabolites

**Figure 11:**
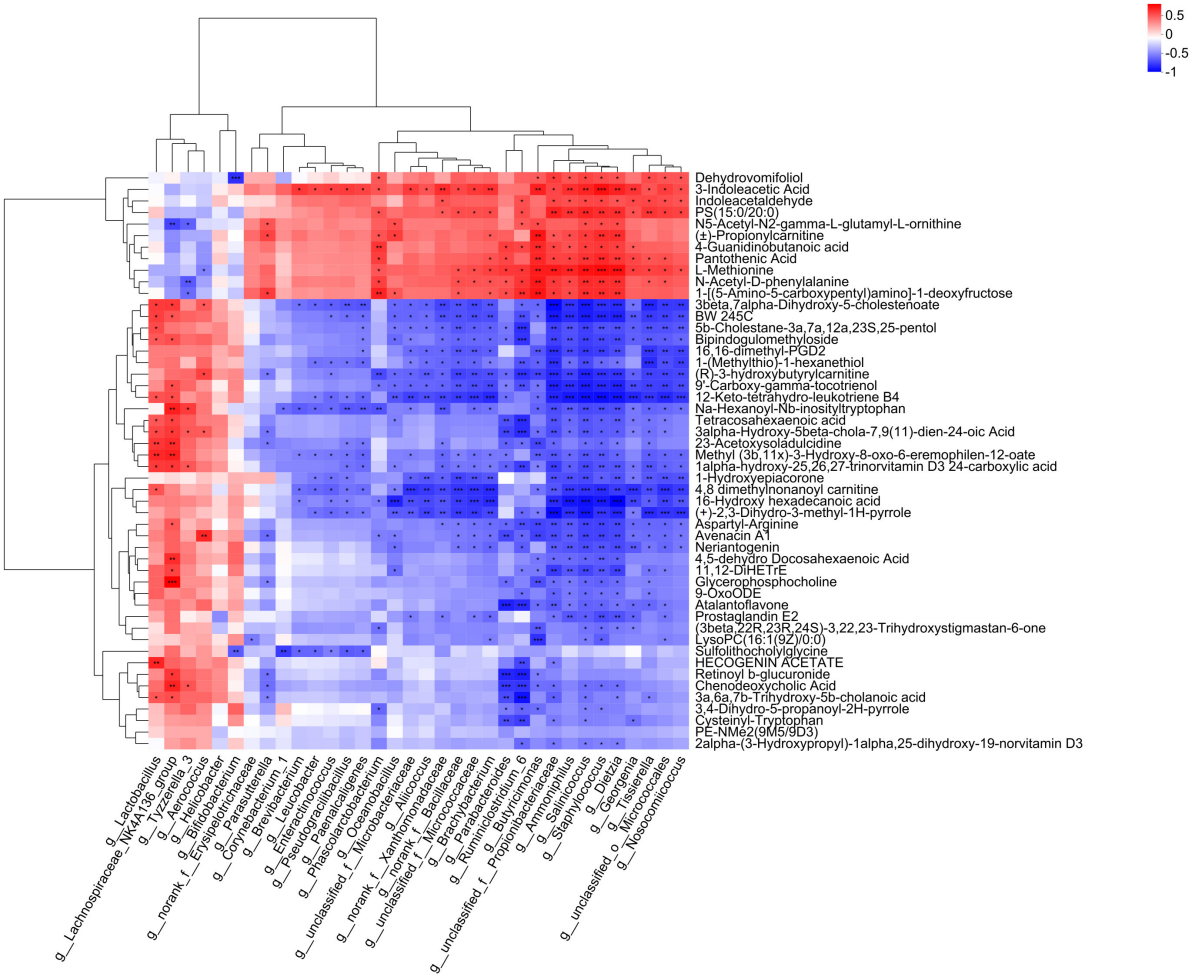
Correlation analysis between gut microbiota and plasma metabolites. The right side of the figure shows the names of plasma metabolites, and the bottom lists the names of the gut microbiota. Each cell in the figure represents the correlation between two attributes (gut microbiota and plasma metabolites), with different colors indicating the strength of the correlation coefficient between the attributes. *P < 0.05, **P < 0.01, ***P < 0.001.

We performed correlation analysis between the differential microbiota and plasma metabolites between groups and generated a heatmap (Figure 11). The results showed that *Lachnospiraceae* and *Tyzzerella* genera were positively correlated with chenodeoxycholic acid (*P* < 0.01, *P* < 0.05), while Bacteroides genus was negatively correlated with chenodeoxycholic acid (*P* < 0.01). *Lachnospiraceae* and *Lactobacillus* genera were positively correlated with 12-keto-tetrahydro-leukotriene B4 (*P* < 0.05), while Bacteroides genus was negatively correlated with 12-keto-tetrahydro-leukotriene B4 (*P* < 0.05). *Lachnospiraceae* genus was positively correlated with glycerophosphocholine (*P* < 0.01), while Bacteroides genus was negatively correlated with glycerophosphocholine (*P* < 0.05), and positively correlated with pantothenic acid and L-methionine (*P* < 0.05).

## Discussion

### taVNS modulates the gut microbiota community composition and function in CUMS rats

In this study, we found that CUMS-induced rats exhibited a series of depressive-like behaviors, including a decrease in open field activity, reduced sucrose preference rate, and an extended immobility time in the forced swimming test. Meanwhile, the composition of gut microbiota in rats underwent significant changes. After taVNS intervention, the gut microbiota of the CUMS rats showed corresponding alterations, and their depressive-like behaviors were significantly suppressed. Previous studies have found that gut microbiota dysbiosis is often present in both depressed human populations and CUMS-induced depressive-like animal models, accompanied by increased levels of inflammatory factors and abnormalities in substance and energy metabolism (Shen et al., 2021; Song et al., 2022; Gong et al., 2024). Dysregulation of the composition and function of the gut microbiota influences the onset and progression of depression through the gut-brain axis (Smet et al., 2022; Chen et al., 2024; Liu et al., 2023). Some studies have found that the Alpha diversity of the gut microbiota is decreased in CUMS mice and rats, and it increases after drug interventions (Tan et al., 2022; Wang X. H. et al., 2024). Our study shows that the Beta diversity of the gut microbiota in CUMS rats significantly differs from the normal group, PcOA analysis showed that the CUMS group had a good discrimination from the blank group. Although the plot shows some overlap between the taVNS group and the CUMS group, it still showed certain discrimination at the OTU level. The analysis of intergroup differences in Beta diversity confirmed the above view from another statistical perspective. The Alpha diversity exhibits a decreasing trend in CUMS group. After taVNS intervention, the Alpha diversity showed a recovery. The Alpha diversity is used to assess the richness of microbial species, while Beta diversity is commonly employed to evaluate the differences or similarities in microbial community structure. Our study suggests that there are no significant differences in microbial richness between the groups, indicating no notable variation in the diversity of microbial species across groups. However, significant differences were observed in the microbial community structure, particularly in the composition of beneficial and harmful bacteria. Specifically, the proportion of beneficial and harmful bacteria varies significantly between these groups. Previous studies have found that Firmicutes, Actinobacteria, and Bacteroidetes are key phyla affecting depression, with an imbalance in the ratio of Bacteroidetes to Firmicutes being observed in patients with depression (Shen et al., 2021; Song et al., 2022). Wei et al. (2019) found that in CUMS rats, the ratio of Firmicutes to Bacteroidetes in the gut microbiota was increased, leading to gut microbiota dysbiosis and subsequently triggering colonic inflammation. Electroacupuncture at Zusanli (ST36) and Tian Shu (ST25) points can suppress CUMS-induced depressive-like behaviors in rats by reducing the ratio of Firmicutes to Bacteroides in the gut microbiota (Wang Z. et al., 2024). Our microbiome characterization index and microbiome profiling analysis also confirmed the above conclusion.

Gut microbiota plays a crucial role in the metabolism of nutrients, the synthesis of vitamins, and the overall metabolic function. Dysbiosis triggered by stress can lead to impaired vitamin digestion and absorption in CUMS rats, which in turn may result in metabolic disturbances in the body, while supplementation with vitamin D and vitamin B can alleviate depressive-like behaviors in the CUMS animal model (Camilleri, 2021; Virili et al., 2018). Biotin, as a coenzyme for vitamin B7, is crucial for maintaining brain metabolism. It also plays an important role in regulating liver glucose uptake, gluconeogenesis, lipogenesis, insulin receptor transcription, and pancreatic β-cell function (Kennedy, 2016). Our study found that biocytin and vitamin D3 levels significantly increased after taVNS intervention, suggesting that taVNS not only improved the vitamin digestion and absorption functions in CUMS rats, but may also concurrently enhance other metabolic functions in the body.

### taVNS suppresses peripheral inflammation mediated by gut microbiota in CUMS rats

The imbalance of gut microbiota leading to intestinal inflammation may result in systemic low-grade inflammation, which is a common feature of mental disorders. Inflammatory cytokines can increase the permeability of the gut and blood-brain barriers, both of which are associated with various psychiatric diseases (Thompson et al., 2023; Severance et al., 2014). Immune activation and the secretion of pro-inflammatory cytokines have gradually been recognized as key mechanisms in the pathogenesis of depression (Beurel et al., 2020; Wang et al., 2017). Our findings also suggest that the gut microbiota dysbiosis in CUMS rats leads to the dysregulation of pro-inflammatory metabolites. Therefore, anti-inflammatory therapies could be a potential treatment strategy.

We further conducted inter-group differential analysis of the microbiota, and found significant distribution differences among Lachnospiraceae_NK4A136_group, *Helicobacter, Lactobacillus*, Tyzzerella_3, norank_f__Erysipelotrichaceae, *Parabacteroide*s, and Corynebacterium_1, all of which showed responses to taVNS intervention. The Lachnospiraceae_NK4A136_group, *Lactobacillus*, and *Tyzzerella* are closely associated with neuropsychiatric disorders. Studies have reported that the abundance of Lachnospiraceae_NK4A136_group increases during the processes of anxiety, depression, and cognitive impairment. Moreover, it has been found to have a significant positive correlation with gut immune factors, including IL-10, TGF-β, IgA, and IL-22 (Xia et al., 2021). Schwarz et al. (2018) found that *Lactobacillus* is negatively correlated with superoxide dismutase (SOD) levels, and it interferes with the metabolism of neuroactive substances, including gamma-aminobutyric acid (GABA), tryptophan, and short-chain fatty acids. This is accompanied by an increase in oxidative stress levels, which may indirectly reflect the pro-inflammatory effects of *Lactobacillus* on clinical phenotypes (Wang J. Y. et al., 2024). Studies have found that *Tyzzerella* is a risk factor for prenatal depression (Fang et al., 2023), and its abundance in the gut is increased eightfold in patients with central nervous system inflammation caused by neurodegenerative diseases (Tremlett et al., 2021).

norank_f__Erysipelotrichaceae, *Parabacteroides*, and Corynebacterium_1 play an active role during the intervention of transcutaneous auricular vagus nerve stimulation (taVNS). Their abundance levels in the gut of rats in the CUMS model were reduced, but increased after the intervention. norank_f__Erysipelotrichaceae and Corynebacterium_1 play an active role in suppressing intestinal inflammation and maintaining gut barrier function (Zhang et al., 2023). The increase in the abundance of norank_f__Erysipelotrichaceae has a causal relationship with a lower risk of inflammatory bowel disease, and it is negatively correlated with inflammatory factors such as TNF-α, while positively correlated with occludin and ZO-1 (Zhuang et al., 2022). The abundance of Corynebacterium is reduced in the CUMS mouse model (Zhao Y. N. et al., 2024). Pang et al. (2024) found that an increase in Corynebacterium abundance can improve the activity and exploratory behavior of mice. Research has shown that the abundance of *Parabacteroides* in the gut of chronic restraint stress mice is reduced, accompanied by an increase in hippocampal inflammatory factor levels. A clinical study found that the level of *Parabacteroides* in the gut of patients with depression is lower than that in healthy individuals, and *Parabacteroides* is negatively correlated with HAMD scores (Guo et al., 2024). Members of *Parabacteroides* promote the expression of the GABA pathway, with GABA being a major inhibitory neurotransmitter that plays a crucial role in stress regulation in the brain. Furthermore, *Parabacteroides* is positively correlated with the metabolites of dopamine (DA) and norepinephrine (NE), including 3-MT, HVA, and MHPG, suggesting that the reduction in *Parabacteroides* abundance may lead to depressive-like behaviors by affecting DA and NE metabolism (Yang et al., 2021). Based on the above research findings, we hypothesize that both the brain neurons and the gut of CUMS rats may exhibit concurrent inflammatory pathological damage. The results of this study suggest that the inflammatory pathological damage in the brain and gut may be alleviated after taVNS. Lachnospiraceae_NK4A136_group, *Parabacteroides*, and Corynebacterium_1 could be potential therapeutic targets for taVNS. Based on the above results, the combination of taVNS with supplements or inhibitors of gut microbiota or its metabolites may enhance the antidepressant therapeutic effects.

Leukotrienes and prostaglandins are metabolic products of arachidonic acid (AA). The key enzymes and metabolites involved in the arachidonic acid metabolic pathway are closely associated with the onset and progression of inflammation (Brash, 2001). 11,12-DiHETrE is generated by the hydrolysis of 11,12-EET by soluble epoxide hydrolase (sEH). Epoxyeicosatrienoic acids (EETs) have anti-inflammatory effects, and 11,12-DiHETrE may indirectly promote the release of pro-inflammatory factors such as Gro-β and COX2 by counteracting the anti-inflammatory effects of EETs (Revermann et al., 2010). 12-keto-TH-LTB4 is a metabolite of the pro-inflammatory mediator leukotriene B4 (LTB4), a potent chemoattractant that promotes neutrophil recruitment and stimulates the release of inflammatory cytokines (Tager et al., 2003). 6-Keto-prostaglandin F1α, as a degradation product of PGI2, exhibits anti-inflammatory effects (Wang et al., 2015). Our study indicates that taVNS can slightly reverse the inflammatory metabolites related to arachidonic acid metabolism in the plasma of CUMS rats. Although the results did not show significant statistical significance, they still indicate that taVNS has certain anti-inflammatory potential. For example, studies have shown that taVNS can significantly reduce the levels of pro-inflammatory factors IL-1β, TNF-α, MCP-1, IL-18, MIP-1α, and MIP-3α in the serum of depressive-like rats, and increase the levels of anti-inflammatory factors IL-4 and IL-10 (Song et al., 2025). This provides strong support for the peripheral anti-inflammatory effect of taVNS. The joint analysis of the gut microbiota and plasma metabolites revealed that pro-inflammatory bacteria such as *Lactobacillus*, Lachnospiraceae_NK4A136_group, *Helicobacter*, and Tyzzerella_3 were positively correlated with pro-inflammatory metabolites in the arachidonic acid metabolic pathway. In contrast, beneficial microbiota like norank_f__Erysipelotrichaceae, Corynebacterium_1, and *Parabacteroides* were negatively correlated with the pro-inflammatory metabolites in this pathway. These findings further support that taVNS can exert a combined regulatory effect on both pro-inflammatory microbiota and inflammatory factors in the arachidonic acid metabolic pathway.

### taVNS suppresses the bile acid and lipid metabolism disorders mediated by the gut microbiota in CUMS rats

Previous studies have reported bile acid and lipid metabolism disorders related to the liver in patients with depression and in the CUMS animal model. The concentration of bile acid in CUMS rat model was abnormally elevated (Goodwin et al., 2000). Our study found that in the CUMS group, chenodesoxycholic acid was positively correlated with harmful microbiota, suggesting that gut microbiota dysbiosis in CUMS rats may lead to bile acid metabolism abnormalities. The results show that chenodesoxycholic acid is positively correlated with *Lactobacillus* and negatively correlated with *Parabacteroides*. Bile acid metabolism can activate the STAT3 inflammatory signaling pathway, leading to the accumulation of *Lactobacillus* in the mouse stomach, which is an important mechanism for the pathological change of gastric inflammation to intestinal metaplasia (Chen et al., 2018). Our preliminary study found that taVNS could alter the phosphorylation levels of STAT3 in the hippocampus and hypothalamus of CUMS rats (Wang J. Y. et al., 2021; Chen et al., 2023). Therefore, it can be inferred that *Lactobacillus* may be involved in the bile acid metabolic pathway, using the gut-brain axis to communicate the expression of pro-inflammatory factors between the peripheral and central systems, thereby damaging brain neurons and triggering a series of psychiatric disorders. Additionaly, the gut-liver axis indirectly influences the onset of depression by modulating bile acid metabolism. As a primary bile acid, chenodesoxycholic acid is converted into secondary bile acids by intestinal microbes expressing bile salt hydrolase (BSH). *Lactobacillus*, which can express BSH, shows an increased abundance in non-alcoholic fatty liver disease (NAFLD), a known independent risk factor for depression. Studies have also shown elevated bile acid levels in the CUMS rat model, which are associated with high expression of FXR, leading to disorders of bile acid metabolism in the liver, intestines, and brain (Wang P. et al., 2025; Qu et al., 2022; Wang W. et al., 2025). Our study suggests that the dysregulation of bile acid metabolism induced by the increased abundance of *Lactobacillus* in CUMS rats may play a crucial role in the pathophysiology of depression. taVNS slightly reduced the plasma levels of chenodesoxycholic acid and bile acids in CUMS rats. Although the results were not statistically significant, taVNS still demonstrates potential therapeutic effects in improving bile acid metabolism.

Studies have found short-chain fatty acids secreted by the Firmicutes phylum are involved in glycerophospholipid metabolism disorders, which in turn disturb the tryptophan pathway and alter brain neurotransmitter levels (Xie et al., 2022). Our study found lipid metabolism disorders in CUMS rats. The gut microbiota significantly influences lipid metabolism in the brain, particularly glycerophospholipid metabolism. Glycerophospholipid metabolism is closely associated with depressive-like behaviors, and gut microbiota dysbiosis may lead to depressive-like behaviors in mice by mediating peripheral and central glycerophospholipid metabolism disorders (Tian et al., 2022). Studies have found that LysoPC (16:1(9Z)/0:0) is decreased in acute liver injury models, leading to lipid metabolism abnormalities, and related pathological processes (Liu et al., 2019). Our study showed that after taVNS intervention, LysoPC [16:1(9Z)/0:0] levels increased, suggesting improvements in liver function and lipid metabolism in CUMS rats.

### taVNS suppresses the amino acid metabolism disorders mediated by the gut microbiota in CUMS rats

Stress-induced depressive-like behaviors are often accompanied by disturbances in amino acid metabolism. Our study found abnormalities in cysteine and methionine metabolism, as well as β-alanine metabolism, in CUMS rats. Methionine is a precursor of S-adenosylmethionine (SAM), which provides methyl groups for various biochemical reactions, including the methylation of key components involved in monoaminergic neurotransmission in depression, thereby exerting an antidepressant effect (De Berardis et al., 2016). Our study found that the methionine levels were reduced in CUMS rats. Other studies have found that the learned helplessness depression rat model exhibits abnormal amino acid metabolism in the prefrontal cortex, particularly in the metabolism of glutamate, cysteine, methionine, arginine, and proline, all of which are significantly disrupted. Notably, both methionine and adenosine levels are reduced (Zhou et al., 2017; Rizzi et al., 2021). Pantothenic acid, in the form of coenzyme A, participates in amino acid metabolism. Moderate intake of pantothenic acid and niacin can reduce the incidence of anxiety. Furthermore, pantothenic acid can alleviate lipid metabolism disorders and inflammation by inhibiting the JNK/P38 MAPK signaling pathway (Mahdavifar et al., 2021; Zhao C. Z. et al., 2024). We found that pantothenic acid and methionine were positively correlated with beneficial gut microbiota and negatively correlated with pro-inflammatory and harmful microbiota. P-Salicylic acid is involved in phenylalanine metabolism. Studies have shown that serum phenylalanine levels increase with the severity of depressive symptoms, making it a potential biomarker for major depressive disorder (Ho et al., 2023). After taVNS intervention, N-Formyl-L-methionine levels increased slightly, P-Salicylic acid was downregulated significantly, and the levels of some other amino acid metabolites were increased significantly, indicating that taVNS can reverse amino acid metabolism disorders in CUMS rats.

## Conclusion

In summary, this study revealed the biological mechanisms of taVNS intervention on the gut microbiota and plasma metabolites in CUMS rats through gut microbiota sequencing and plasma metabolomics. The strength of this study lies in its use of a multi-omics approach, which supplements and enriches the understanding of depression, aiding in the identification of potential therapeutic targets for developing new treatments. However, this study also has certain limitations. First, there is a lack of biological experimental validation for the molecular markers. Future research should further focus on the gut-brain barrier system and design relevant experiments. By severing the vagus nerve, the inflammatory states in the gut and central nervous system can be observed and the role of the vagus nerve in regulating the crosstalk between peripheral and central inflammation during the anti-inflammatory and antidepressant process of taVNS. Second, there are still differences between the gut microbiota and plasma metabolites of animals and humans, necessitating further high-quality clinical cohort studies to reveal the omics characteristics of depression in humans and the intervention effects of taVNS. Third, future studies should incorporate larger sample sizes and employ advanced bioinformatics algorithms to enhance data robustness. In conclusion, this study systematically analyzed the effects of taVNS on the gut microbiota, plasma metabolites, and their enriched pathways in CUMS rats, providing a theoretical foundation and guidance for further research into the potential mechanisms of taVNS in treating neuropsychiatric disorders.

## Data Availability

The data presented in the study are deposited in the Sequence Read Archive (SRA), accession number PRJNA1281334.
